# Echocardiographic findings in patients with acute pulmonary embolism at Sohag University Hospitals

**DOI:** 10.1186/s43162-022-00114-y

**Published:** 2022-02-19

**Authors:** Mohamed Eid, Ahmed Mohamed Boghdady, Mustafa Mohamed Ahmed, Lotfy Hamed Abu Dahab

**Affiliations:** Department of Internal Medicine, Faculty of Medicine, Sohag University, Sohag, Egypt

**Keywords:** Acute PTE, Echocardiography, Tricuspid regurge, Pulmonary hypertension, McConnell’s sign, RV dilatation, RV thrombosis

## Abstract

**Background:**

Acute pulmonary thromboembolism (PTE) is one of the serious medical issues with higher prevalence and mortality rates. As mentioned in several medical reports, most of the chest pain patients, visiting the emergency departments, are usually diagnosed with either acute PTE, acute coronary syndromes, or acute aortic syndromes. The current study aimed to study the risk factors and explore the echocardiographic findings in patients with PTE.

**Results:**

Forty patients with acute pulmonary embolism were enrolled in the study. Echocardiography and computed tomography pulmonary angiography (CTPA) were evaluated for all participants. The echocardiography showed that 29 patients (72.5%) had echocardiographic findings suggestive of acute PTE. Twenty-four patients (60%) had tricuspid regurge. Twenty-one patients (52.5%) had dilated right ventricle (RV). Also, 13 patients (32.5%) had an echocardiographic finding of pulmonary hypertension. Furthermore, ten patients (25%) had McConnell’s sign, and 21 patients (52.5%) had RV systolic dysfunction where only two (5%) showed RV thrombosis. Echocardiographic data of the eight high-risk patients showed that 6 patients (75%) had TR, 8 patients (100%) had dilated RV, 5 patients (62.5%) had pulmonary hypertension, 8 patients (100%) had McConnell’s sign, one patient (12.5%) had RV thrombus, and 8 patients (100%) had RV systolic dysfunction.

**Conclusion:**

The results revealed that thrombus in the main pulmonary trunk was a high-risk factor for patients with acute pulmonary embolism. The current study suggested that echocardiography is an important bedside imaging tool for the diagnosis of PTE. Echocardiography could detect the tricuspid regurge, pulmonary hypertension, McConnell’s sign, RV dilatation, thrombosis, and dysfunction. Furthermore, echocardiography was considered a non-invasive test for rapid diagnosis of PTE and determining the degree of the risk category (high- or low-risk patients) specially with the presence of McConnell’s sign, dilated RV, and RV systolic dysfunction.

## Background

Acute pulmonary thromboembolism (PTE) is one of the life-threatening diseases with higher incidence and mortality rates [1]. In the USA, more than 600,000 cases are diagnosed with PTE annually, with a calculated and expected mortality rate of 50,000–200,000 PTE-related deaths per year [2]. Worldwide, the overall 3-month mortality rate of all PTE patients is almost 15%, and 50% of them had a shock [1]. The reported mortality rates of PTE exceeded some of the other life-threatening diseases, such as acute myocardial infarction [3]. Besides, the prevalence of PTE had increased after the pandemic disaster of COVID-19 disease, in 2020, with increased mortality rates [4]. In the last four decades, the overall mortality rate of PTE was declining in Europe while in the USA was relatively stable despite the recent development in the diagnosis and screening tools of that disease [5].

According to different records from emergency departments, worldwide, PTE is one of the most diagnosed diseases for patients with chest pain, besides acute coronary and acute aortic diseases [6]. The most reported symptoms of PTE include chest discomfort and dyspnea in most cases; however, these symptoms are similar for PTE and acute coronary syndromes, as well [7]. Based on the physical diagnosis, the clinical features of PTE are not easily differentiated, which prompts physicians, especially cardiologists, to search for more accurate diagnostic tools [6]. One of the important tools for PTE diagnosis is the transthoracic echocardiography (TTE), which is a non-invasive model that can provide bedside results [8]. It is considered one of the valid tools to estimate the risk stratification in PTE patients [9].

It also allows the adequate imaging and screening of the main blood vessels, such as the aorta and the left ventricle, which might evaluate any other causatives of angina and that facilitates PTE prognosis prediction [10]. Currently, RV dilatation or dysfunction is the most common indicator for thrombolytic therapy, despite its poor prognosis [11]. Furthermore, tricuspid annular plane systolic excursion (TAPSE) as a measurement for RV systolic dysfunction was decreased in acute PTE patients and was independently foretelling poor prognosis [12]. The electrocardiographic changes are indicatives of RV strain, which might be helpful tools for PTE diagnosis. These changes include the abnormal T waves in leads V1–V4, a QR pattern in V1 which is an important ECG sign of PTE, incomplete or complete right bundle-branch block (RBBB), and the resulted S1Q3T3 pattern [13]. These ECG changes are dependent on the severity level of PTE, as despite all changes that will occur with severe PTE, only sinus tachycardia in 40% of patients with milder PTE [14]. Furthermore, atrial arrhythmias or the so-called atrial fibrillation might be associated with acute PTE, as well [15]. One of the most common features in acute PTE patients with TTE is the McConnell’s sign, as it shows normal contraction or sparing of the RV apex with hypokinesis of the midportion of the RV-free wall [16].

The current study aimed to investigate the risk factors of PTE and to highlight the importance of echocardiographic imaging in clinical diagnosis.

## Patients and methods

### Patients

The current observational single-center study was conducted in the Coronary Care Unit at Sohag University Hospitals, Sohag, Egypt, in the period between May 2017 and May 2018. Forty patients were enrolled in the study, where all of them were admitted to the coronary care unit with acute pulmonary embolism which was confirmed by computed tomographic pulmonary angiography. All patients with acute pulmonary embolism (with age ≥ 18 years) were included in the study. Otherwise, younger patients and those with specific clinical circumstances, such as kidney failure (on dialysis), pregnancy, malignancy, and other severe comorbidities, were excluded. Besides, patients diagnosed with chronic chest, rheumatic, or ischemic heart diseases were excluded because of the similarity of symptoms with PTE.

The study was assessed by the Scientific and Ethical committees of Sohag Faculty of Medicine according to the guidelines of the Helsinki Declaration for human research studies available from https://www.wma.net/. All the written consents were collected from all participants in the current study.

### Study design

The patients were divided into two groups according to their systolic blood pressure (SBP):

- Group A: high-risk category, diagnosed by shock or hypotension (SBP ≤ 90 mmHg)
- Group B: low-risk category, not diagnosed with shock or hypotension

For all participants, the data of age and gender were collected. Data of other clinical features and medical histories, such as diabetes milletus (DM), hypertension, deep venous thrombosis (DVT), surgical operations, or any other co-morbidities, were included, as well.

### Laboratory investigations

To confirm the clinical profile of the studied population, different laboratory investigations such as the complete blood counting (CBC), levels of fasting blood sugar (FBS), lipid profile, coagulation profile, renal and liver function tests, and levels of cardiac troponin were measured. Besides, the d-dimer testing was performed for all participants and was considered positive (> 500 ng/dl) in patients below 50 years, and in patients with age above 50 years, age-adjusted d-dimer (age × 10 mcg/l) value is considered [17].

### Arterial blood gases (ABG)

This test is used to determine the level of blood acidity by measuring the levels of oxygen and carbon dioxide in the arterial blood. The normal partial pressure of oxygen (PaO_2_) is equal to 80–100 mmHg. Hypoxemia is defined as PaO_2_ less than 80 mmHg. It is further graded as mild hypoxemia (PaO_2_ = 60–80 mmHg), moderate hypoxemia (PaO_2_ = 40–60 mmHg), and severe hypoxemia (PaO_2_ < 40 mmHg) [18]. Hypocapnia is defined as a decrease in blood carbon dioxide level below the normal reference range of 35 mmHg [18]. Hypocapnia is usually caused by conditions causing hyperventilation. ABG shows if there is hypoxemia, hypocapnia, or both in the same patient.

### Computed tomography pulmonary angiography (CTPA)

CTPA is a common CT scan that is used to screen blood clots (the thrombus) or pulmonary embolism in the lung’s arteries [19]. All CTPA studies were done on Toshiba’s Alexon (a 16-row detector CT scanner) with intravenous administration of iodinated contrast material (OMNIPAQUE 350/50 ml) at 3–5 ml/s with timing optimized for the pulmonary artery using bolus tracking and automatic triggering. CTPA was done as soon as possible or after quick resuscitation and thrombolysis in high-risk patients. Two well expert radiologists independently reviewed the CT pulmonary angiography-positive examinations on clinical picture archive and communication system monitors.

### Echocardiography

The echocardiography was performed with Toshiba instruments, Japan (Nemio SSA-550A), with a 2.5-MHz transducer and harmonic imaging at the Internal Medicine Department Echocardiography Laboratory. Tricuspid annular plane systolic excursion (TAPSE) was calculated from M-mode through the lateral tricuspid annulus by calculating the amount of longitudinal motion of the annulus at peak systole. TAPSE was estimated as an echocardiographic measure of right ventricular function, and a value less than 17 mm suggests RV dysfunction [20]. Regional right ventricular dysfunction is detected by the 2D mode. Regional right ventricular dysfunction is defined as normal contraction and “sparing” of the right ventricular apex despite moderate or severe right ventricular free-wall hypokinesis, which is known as the McConnell’s sign [16]. The 2D mode was used to measure the right ventricle size from four standardized transthoracic views. Their normal values were as follows; (a) proximal outflow tract parasternal long axis view, > 30 mm abnormal, normal range 20–30 mm; (b) proximal outflow tract parasternal short axis view, > 35 mm abnormal, normal range 21–35 mm. (c) distal outflow tract, > 27 mm abnormal, normal range 17–27 mm; and (d) apical right ventricle at base, > 41 mm abnormal, normal range 25–41 mm. For the apical right ventricle at mid-level, abnormal is > 35 mm, and the normal range is 19–35 mm [21]. Pulmonary arterial hypertension was defined as a mean pulmonary arterial pressure of 25 mmHg or more as measured indirectly by echocardiography. The pulmonary artery systolic pressure can be estimated by measuring the peak velocity of the tricuspid regurgitant jet obtained with Doppler echocardiography. The gradient across the tricuspid valve can be estimated by using the modified Bernoulli equation, *P* = 4V2; *P* represents the peak pressure difference between the right atrium and right ventricle, and *V* is the peak velocity of the regurgitant jet. Estimated right atrial pressure is added to the gradient to estimate the pulmonary artery systolic pressure [22]. The following formula was derived to estimate mean pulmonary artery pressure: mean PAP = 0.65 PASP + 0.55 mmHg.

### Statistical analysis

Data were analyzed using STATA intercooled version 14.2 (StataCorp, College Station, TX, USA). Quantitative data were represented as mean, standard deviation, median, and range. The Mann-Whitney test was used to compare the two groups. Qualitative data or frequencies of different variables were tested and compared with either the chi-square or Fisher exact tests. *P* value < 0.05 was considered significant.

## Results

In the current study, 40 participants were recruited and included 24 females and 16 males with an age range from 24 to 59 years. The medical history and risk factors of the study population are shown in Table 1. About seven patients (17.5%) were diabetic, while four patients (10%) suffered from hypertension. As regards the risk factors, idiopathic PTE was found in 9 patients (22.5%), and six patients (15%) had a recent surgical operation. DVT was found in 24 patients (60%), one patient (2.5%) had a history of cancer, and three (7.5%) had thrombophilia, where only three patients (7.5%) received contraceptive pills.

**Table 1:** Clinical characteristics and risk factors of the studied populations

**Clinical characteristics**	
**Age/years (range)**	24–59
**Male sex**	16 (40%)
**Diabetes mellitus (DM)**	7 (17.5%)
**Hypertension**	4 (10%)
**Risk factors**	
**Abdominal pelvic or orthopedic surgical operation**	6 (15%)
**History of DVT**	24 (60%)
**Treated with contraceptives**	3 (7.50%)
**Cancer**	1 (2.5%)
**Thrombophilia**	3 (7.5%)
**Idiopathic**	9 (22.5%)

The results of echocardiography revealed that 11 patients (27.5%) from the 40 patients had no significant findings suggestive of PTE while 29 patients (72.5%) had PTE echocardiographic findings. Twenty-four patients (60%) had tricuspid regurge, 21 patients (52.5%) had dilated RV, 13 patients (32.5%) had an echocardiographic finding of pulmonary hypertension, ten patients (25%) had McConnell’s sign, 21 patients (52.5%) had evidence of RV systolic dysfunction, and only two patients (5%) had RV thrombosis, as shown in Table 2.

**Table 2:** Echocardiographic findings of the studied patients

Finding	Frequency, ***N*** (%)	***P*** value
**Tricuspid regurge**	24 (60%)	0.321
**Pulmonary hypertension**	13 (32.5%)	*0.049
**Dilated RV**	21 (52.5%)	*0.000
**RV thrombosis**	2 (5%)	*0.000
**McConnell’s sign**	10 (25%)	0.329
**RV systolic dysfunction (TAPSE)**	21 (52.5%)	*0.000

*Significant *P* value < 0.05

Furthermore, the clinical examination of the studied population revealed that all of the 40 participants had dyspnea, 18 patients (45%) had chest pain, eight patients (20%) were hypotensive, and only four patients (10%) had hemoptysis. ABG test results revealed that 24 patients (60%) were hypoxic, where 12 patients (30%) were hypoxic and hypocapnic. All of the patients below 50 years had higher levels of d-dimer (> 500 ng/dl). Patients ≥ 50 years had d-dimer values above the age-adjusted d-dimer value (age × 10 mcg/l) (Table 3). The different laboratory investigations showed relevant expected results for each participant according to his clinical profile.

**Table 3:** Relation between the presence of risk stratification of PTE and different investigations

Variable	High risk, ***n*** = 8	Low risk, ***n*** = 32	***P*** value
ECG			
Tachycardia	4 (50.00%)	13 (40.63%)	0.89
Tachycardia + SQT	3 (37.50%)	14 (43.75%)	
RV strain pattern	1 (12.50%)	5 (15.62%)	
ABG			
Normal	0 (0%)	4 (12.50%)	0.55
Hypoxia	5 (62.50%)	19 (59.38%)	
Hypoxia and hypocapnia	3 (37.50%)	9 (28.12%)	
d-dimer			
Normal (< 500 ng/dl)	0 (0%)	0 (0%)	1.00
Positive (> 500 ng/dl in pts < 50 years, and above age-adjusted d-dimer values in pts ≥ 50 years)	8 (100%)	32 (100%)	

**Fig. 1:**
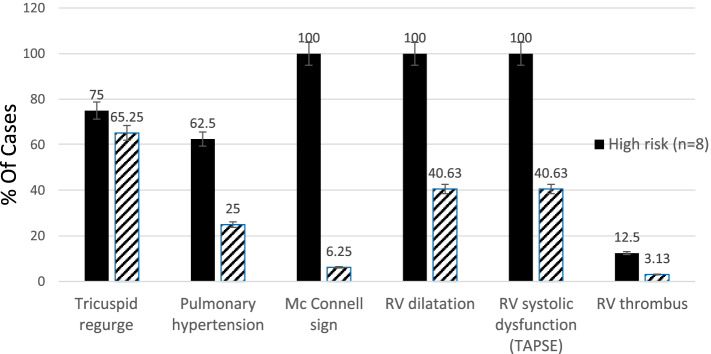
Relation between risk stratification of PTE and echocardiographic findings

According to the SBP results, only eight patients (20%) were considered at high-risk (group A), where 32 patients (80%) had a lower risk. Further analysis of the echocardiography results according to risk groups revealed that pulmonary hypertension, McConnell’s sign, dilated RV, and RV systolic dysfunction as estimated as TAPSE < 17 mm were significantly more in the high-risk category (group A) (Table 4, Fig. 1).

**Table 4:** Echocardiography finding of both risk groups of the studied populations

Echocardiographic findings	Total	High risk (***n*** = 8)	Low risk (***n*** = 32)	***P*** value
**Tricuspid regurge**	24	6 (75%)	18 (56.25%)	0.333
**Pulmonary hypertension**	13	5 (62.5%)	8 (25%)	0.043
**McConnell’s sign**	10	8 (100%)	2 (6.25%)	*0.000
**RV dilatation**	21	8 (100%)	13 (40.63%)	*0.003
**RV systolic dysfunction**	21	8 (100%)	13 (40.63%)	*0.003
**RV thrombus**	2	1 (12.5%)	1 (3.13%)	0.277

*Significant *P* value < 0.05

**Fig. 2:**
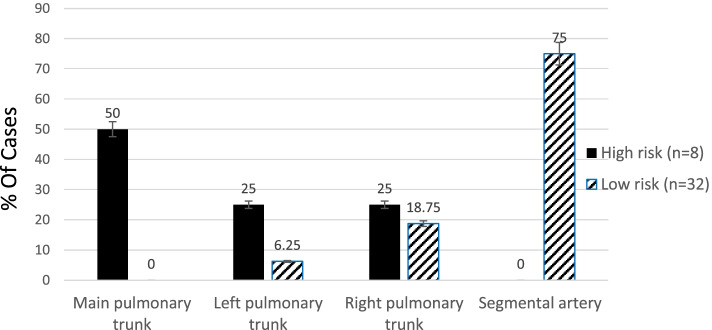
Relation between risk stratification of PTE and the site of thrombus

It was noticed that thrombus in the main pulmonary trunk was found in four patients, all of them were in the high-risk group, while left pulmonary artery thrombus was found in four patients, two of them were in the high-risk group (25%) and the other two were in the low-risk group (6.25%). Furthermore, thrombus in the right pulmonary artery was found in eight patients, two in the high-risk group (25%) and six in the low-risk group (18.75%). Thrombus in the main pulmonary trunk has a significant relationship with the high-risk category (group A), as shown in Table 5 and Fig. 2. The results of CTPA (Fig. 2) showed that, according to the thrombus site, about 16 patients (40%) had thrombus occluding the main pulmonary trunk, and left and right pulmonary arteries while 24 patients (60%) had thrombus at the segmental level of the pulmonary arteries.

**Table 5:** Relation between the site of thrombosis and risk stratification of PTE

Site of thrombus	Total	High risk (***n*** = 8)	Low risk (***n*** = 32)	***P*** value
**Main pulmonary trunk**	4	4 (50%)	0 (0%)	*0.000
**Left pulmonary trunk**	4	2 (25%)	2 (6.25%)	0.114
**Right pulmonary trunk**	8	2 (25%)	6 (18.75%)	0.693
**Segmental artery**	24	0 (0%)	24 (75%)	*0.000

*Significant *P* value < 0.05

The statistical analysis did not reveal any significant relationship between the risk stratification (high-risk and low-risk patients) and the risk factors such as DM, hypertension, history of DVT, and history of operation (Table 1), or the investigations (Table 3), where for CTPA, the site of thrombus had a significant relationship with the high-risk category (Table 5). Besides, the thrombus in the main pulmonary trunk was found to be highly associated with the high-risk patients, as well (Table 5 and Fig. 2).

## Discussion

The presentation varies from one patient to another. All of them had dyspnea, 18 patients (45%) had chest pain, eight patients (20%) were hypotensive, and only four patients (10%) presented with hemoptysis. This is similar to the findings of the study conducted by Kostrubiec and colleagues [23].

ECG is one of the important tools to suspect and diagnose pulmonary embolism, especially in the presence of tachycardia without an apparent cause, which are in an agreement with the previous findings of Eichinger and colleagues [24]. ECG finding differs according to the severity of the condition about 17 patients (42.5%) had tachycardia, 17 patients (42.5%) have tachycardia with S1Q3T3 pattern, and about six patients (15%) with RV strain. The abovementioned ventricular strain patterns induced changes in ECG [13] which are unique to each patient according to his/her clinical and social characteristics.

Echocardiography in the current study revealed that 27.5% of patients had no significant findings suggestive of PTE. These findings were near similar to previous study results that showed that 71% of 511 patients confirmed to have acute PTE had no significant findings of acute PTE [25]. 72.5% of the studied patients had the following echocardiographic findings suggestive of acute PTE: 24 patients (60%) have tricuspid regurge, 21 patients (52.2%) have dilated right ventricle, 13 patients (32.5%) have pulmonary hypertension, ten patients (25%) have McConnell’s sign, 21 patients (52.5%) had RV systolic dysfunction, and only two patients (5%) had RV thrombosis. In an agreement with these findings, a previous study showed that 90% of PTE patients had tricuspid regurge, 75% had dilated RV, 77% had pulmonary hypertension, 25% had McConnell’s sign, 20% had RV systolic dysfunction, and only 4% had RV thrombosis [26, 27]. Also, several previous studies agreed with our findings [24, 25, 28–30]. This might be due to the same effect of pulmonary embolism on the heart. Our study also revealed that McConnell’s sign, dilated RV, and RV systolic dysfunction as assessed by TAPSE had a significant relation to high-risk patients with acute PTE. This agreed with previous studies which identified the RV dilatation and TAPSE as a measurement of RV dysfunction, to be independently associated with more hemodynamic instability and mortality in patients presenting with acute PTE [12, 31, 32]. In contrast to our results, Dahhan et al. found in their retrospective study that RV systolic dysfunction measured by TAPSE was not associated with mortality on univariable analysis in contrast to RV Tei Index analysis [33]. This may be explained that his study was retrospective, and there was no set protocol for RV imaging while our study was prospective and we already defined TAPSE but not the RV Tei Index as a method for measuring RV systolic dysfunction. The CTPA finding revealed that thrombus in the main pulmonary trunk had a direct proportion with high-risk patients. Similarly, a previous study used CTPA and showed that patients with central PTE had a higher mortality rate of 40% compared to segmental or subsegmental PTE [34].

## Conclusion

The current study revealed that echocardiography is an important bedside imaging tool for the diagnosis of PTE by detecting the following findings as RV dilatation, pulmonary hypertension, RV systolic dysfunction, and McConnell’s sign. The CTPA results suggested that thrombus in the main pulmonary trunk had a direct proportion with high-risk patients.

CTPA is still the gold standard test for the diagnosis of PTE. Despite echocardiography is not a conclusive tool for the diagnosis of PTE, it is an excellent and non-invasive test for rapid diagnosis specially in high-risk patients by defining whom having RV systolic dysfunction, RV dilatation, and McConnell’s sign.

## Data Availability

The data used and/or analyzed during the current study are available from the corresponding author on reasonable request.

## Abbreviations

CBC: Complete blood counting
CTPA: Computed tomography pulmonary angiography
DVT: Deep venous thrombosis
ECG: Electrocardiograph
FBS: Fasting blood sugar
PTE: Pulmonary thromboembolism
RBBB: Right bundle-branch block
RV: Right ventricle
SQT: Short Q-T syndrome
TAPSE: Tricuspid annular plane systolic excursion
TTE: Transthoracic echocardiography
SBP: Systolic blood pressure
